# Complex impact of hypomagnetic fields on yeast growth, volatile components emissions, and cell surface morphology

**DOI:** 10.1128/spectrum.02677-26

**Published:** 2026-09-29

**Authors:** Miroslava Sincak, Hana Brinkeova, Ludek Bartoš, Jaromír Hradecký, Jakub Dušek, Kateřina Benediktová, Jana Sedlakova-Kadukova

**Affiliations:** 1 Faculty of Forestry and Wood Sciences, Czech University of Life Sciences Prague 48371, Suchdol, Czechia; 2 Institute of Animal Science, Praha, Czechia; 3 ALGAJAS s.r.o., Košice, Slovakia; Connecticut Agricultural Experiment Station, New Haven, Connecticut, USA

**Keywords:** yeast, hypomagnetic field, growth kinetics, volatile emission, cultivation volume

## Abstract

The geomagnetic field is a persistent environmental condition under which biological systems have evolved. Its absence creates hypomagnetic conditions, potentially disrupting this equilibrium. This study examined the effects of hypomagnetic fields on *Saccharomyces cerevisiae* growth and metabolism across three culture volumes (10, 75, and 250 mL). Growth inhibition was most pronounced in the smallest volume, with up to 42% reduction in 10-mL cultures, decreasing to 24% in 75 mL, and none in 250-mL cultures. Budding analysis showed significant early inhibition in 10-mL cultures at 2, 4, and 6 h, and in 75-mL cultures at 6 h, whereas no significant budding inhibition was detected in 250-mL cultures. These results suggest that smaller volumes amplify the impact of hypomagnetic exposure, whereas larger volumes may buffer it. These shifts indicate that hypomagnetic exposure affects growth kinetics and extracellular volatile profiles without causing visible cell-surface damage detectable by scanning electron microscopy (SEM), although the underlying molecular mechanism remains unresolved. Our findings identify cultivation volume as an important modifier of microbial sensitivity to magnetic-field deprivation and have implications for interpreting microbial responses in magnetically altered environments, including space-biology models, shielded structures, and fermentation-based systems.

**IMPORTANCE:**

Microorganisms are commonly studied under controlled temperature, medium, and aeration conditions, but the magnetic field environment is rarely considered as an experimental variable. This study shows that short-term hypomagnetic exposure alters growth kinetics and volatile metabolic output in *Saccharomyces cerevisiae*. Importantly, the magnitude of the response depended on cultivation volume: growth inhibition was strongest in small static batch cultures and was not statistically detectable in the largest volume tested. These findings identify cultivation volume as an important modifier of microbial sensitivity to magnetic field deprivation, probably through its influence on culture physiology, gas exchange, metabolite accumulation, and growth phase. The study therefore has implications for the design and interpretation of microbial magnetic-field experiments, including work performed in shielded environments, space biology models, and fermentation-related systems.

## INTRODUCTION

The Earth’s geomagnetic field (GMF), a natural electromagnetic field of approximately 50 μT, has been a constant presence throughout the evolutionary history of life, shaping the biological processes of all organisms (1). Changes in this natural magnetic field can be perceived by organisms at various levels and may influence specific metabolic or physiological processes (2).

Currently, many studies focus on the impact of strong to moderately strong electromagnetic and magnetic fields, but only a limited number explore what happens when the magnetic field is absent or significantly weakened (3, 4). This condition is referred to as a hypomagnetic field (or almost zero field) and is typical of interplanetary space as well as some planets in the solar system (5). Additionally, hypomagnetic fields can be found in urban environments, such as in buildings constructed with steel (6). Understanding microbial responses to such conditions is therefore relevant not only for space-biology research but also for interpreting microbial physiology in magnetically altered terrestrial environments.

Most hypomagnetic field studies have focused on plants and animals, where effects have been reported on cellular respiration (7), ATP production (8), plant growth (9, 10), and neurophysiological or behavioral outcomes such as memory and cognitive performance (5, 11). In contrast, microbial responses to hypomagnetic exposure remain poorly characterized. Although microorganisms have been extensively studied under other space-relevant stressors, including radiation and microgravity (12), direct studies of hypomagnetic effects in microbial systems are limited and have mainly addressed antibiotic tolerance (13) or magnetosome formation in magnetotactic bacteria (14). Yeast, despite its importance as a model eukaryotic microorganism and fermentation organism, has therefore remained largely unexplored in this context.

*Saccharomyces cerevisiae*, or baker’s yeast, is a widely employed model organism across numerous biological disciplines due to its simplicity, rapid growth, and well-characterized genome (15). Studies on the effects of magnetic fields on yeast have revealed varied outcomes. Strong static magnetic fields (100–300 mT) have been shown to induce metabolic changes that influence subcellular functions (15, 16), affect DNA integrity, and cause genetic alterations (17). While many studies report positive effects on yeast growth and viability (18), others document adverse effects (19, 20), and some report no significant changes (21). Changes in growth, proliferation, and cellular respiration under strong magnetic fields are often attributed to oxidative stress (18).

Despite these findings, the effects of hypomagnetic fields remain poorly understood, and current theories explaining microbial magnetoreception of hypomagnetic fields (HMFs) remain unclear and contradictory. Some studies propose that nonspecific physical mechanisms, such as the interaction of magnetic fields with paramagnetic ions or magnetic particles, might play a role (6, 22). Magnetic fields have also been shown to affect metal ions by altering electron spin configurations and the metal-ligand coordination environment, thereby influencing downstream biochemical events (23, 24).

The present study investigated whether short-term hypomagnetic exposure alters growth kinetics and volatile metabolic output in *Saccharomyces cerevisiae*. A specific focus was placed on cultivation volume because static batch cultures of different volumes differ in surface-area-to-volume ratio, passive gas exchange, metabolite accumulation, and the timing of transition toward oxygen-limited or fermentative growth. These culture-level factors are central to yeast physiology but have rarely been considered as modifiers of microbial responses to magnetic-field deprivation. We therefore tested whether cultivation volume modulates the magnitude of the yeast response to hypomagnetic exposure. Because no specific molecular receptor for hypomagnetic-field sensing has been identified in unicellular eukaryotes, the study focuses on reproducible physiological and metabolic outcomes rather than on intracellular signaling mechanisms.

## MATERIALS AND METHODS

### Model organism

To investigate the effect of magnetic fields, we used a strain of yeasts, *Saccharomyces cerevisiae* (CCY 21–4–13, distillery culture), obtained from the collection of the Chemical Institute of the Slovak Academy of Sciences (SAV) in Bratislava, Slovakia. The yeasts were cultivated in liquid YPD medium, provided by Sigma-Aldrich (United States).

### Electromagnetic field setup

A pair of Helmholtz coils in each chamber, combined with a triaxial magnetic-field compensation system (Stefan Mayer Instruments GmbH & Co. KG, Germany), was used to generate controlled static magnetic field conditions. Each chamber consisted of an aluminum-shielded enclosure of approximately 2 m × 2 m × 2 m positioned within the three-axis coil system. Samples containing *Saccharomyces cerevisiae* were placed at the center of the chamber, where the magnetic field is spatially homogeneous.

In the hypomagnetic treatment, the ambient geomagnetic field was actively compensated along the X, Y, and Z axes. In the control treatment, the local geomagnetic field was simulated at approximately 49 μT. Magnetic field values were monitored by an integrated Hall probe positioned close to the sample area. Because field compensation was not absolute, low-intensity external fluctuations and internal sensor noise remained detectable, resulting in a residual magnetic flux density of approximately 0.1 μT under hypomagnetic conditions. Therefore, this treatment is referred to as hypomagnetic rather than zero-field exposure.

The experiment was performed in two consecutive chamber assignments to reduce chamber-specific effects. In Experiment 1, one chamber generated the hypomagnetic condition, and the second chamber generated the geomagnetic control. In Experiment 2, the chamber assignments were exchanged. The magnetic field parameters for both assignments are shown in Table 1.

**TABLE 1:** Electromagnetic chamber setting^*a*^

Experiment	Light conditions (light:dark)	Coil/EMF	F (μT)	X-axis (μT)	Y-axis (μT)	Z-axis (μT)	H (μT)	I (°)
Experiment 1	Dark 0:24	Coil 1	0.1	0.0	0.0	−0.1	0.0	–
	Dark 0:24	Coil 2	49.4	−19.8	1.6	−45.2	19.9	66
Experiment 2	Dark 0:24	Coil 1	49.4	−19.8	1.6	−45.2	19.9	66
	Dark 0:24	Coil 2	0.1	0.0	−0.1	0.0	0.1	–

^*a*^ F, absolute magnetic flux density; X, north-south component; Y, east-west component; Z, vertical component; H, horizontal intensity; I, inclination; –, not applicable for the hypomagnetic treatment because the geomagnetic vector was actively compensated and only a near-zero residual magnetic field remained.

### Experimental design

The yeasts were cultured in liquid YPD medium (1% yeast extract, 2% peptone, 2% glucose) for 2 h. After pre-cultivation, aliquots of the same starting culture were transferred into culture vessels containing 10, 75, or 250 mL of medium. For each volume, one vessel was exposed to hypomagnetic conditions, and one vessel was exposed to the geomagnetic control field, resulting in six vessels per experimental run. The exposure lasted 24 h. The complete experiment was repeated three times independently using freshly prepared cultures, resulting in three biological replicates for each volume × magnetic field condition. Cultivation took place in the dark at controlled laboratory temperature (24°C ± 1°C). No agitation or active aeration was applied during the exposure period; therefore, oxygen availability depended solely on passive diffusion from the headspace. Under these static batch conditions, dissolved oxygen is expected to decrease progressively due to yeast metabolic activity, resulting in predominantly microaerobic to anaerobic conditions. Although dissolved oxygen concentration and redox potential were not directly measured, all volumes were cultivated under identical environmental conditions. Differences between volumes therefore reflect intrinsic variations in surface-area-to-volume ratio, gas exchange dynamics, and potential gradients in dissolved gases and metabolites.

### Growth and pH measurements

Yeast growth was measured by optical density (OD600) every 2 h for 24 h (excluding overnight periods) using a UV–Vis spectrophotometer (WPA Biowave 3).

The initial pH of the freshly prepared YPD medium was 6.5 ± 0.2, consistent with the standard formulation. The medium was not additionally buffered, and no further pH adjustments were performed during cultivation. Changes in pH were measured using a multimeter (WTW inoLab 9630). Measurements were performed after culturing the initial culture for 18 h and after 24 hours of exposure to the respective magnetic field conditions.

During batch cultivation, the pH decreased from the initial value (~6.5) to a range typically between 4.0 and 4.8, reflecting organic acid production and fermentative metabolism under static conditions. No medium supplementation was applied during the 24-h cultivation period.

### Budding analysis

Budding frequency was evaluated as an indicator of early proliferative development. At 0, 2, 4, 6, 23, and 24 h of cultivation, aliquots were collected from each culture volume and magnetic field condition. Cells were examined microscopically, and the percentage of budding cells was calculated as the number of cells with visible bud emergence divided by the total number of counted cells. For each sample, 100 cells were counted. Budding inhibition under hypomagnetic exposure was expressed as the relative percentage change compared with the corresponding geomagnetic control at the same time point and cultivation volume.

### Statistical analyses

Associations between the dependent variable, the yeast growth, and the fixed effects were analyzed using a multivariate general linear model (GLM, PROC MIXED, SAS, version 9.4) designed for repeated measurements (REPEATED = Time, SUBJECT = ID of the yeast sample). The fixed effects were time (0, 2, 4, 6, 23, and 24 h since the start of the yeast cultivation) nested within the strength of the magnetic field (hypomagnetic field and normal magnetic conditions) and volumes (10, 75, and 250 mL). Additionally, the strength of the magnetic field was nested within the volumes. Least-squares means (LSMEANs) were computed for each class, and differences between classes were tested using a *t*-test. We used a Tukey-Kramer adjustment for multiple comparisons. Budding frequencies were compared between hypomagnetic and geomagnetic control conditions within each cultivation volume and time point using a paired *t*-test. Differences were considered statistically significant at *P* < 0.05.

### Volatile emission analysis

Differences between the magnetically affected sample and the control were investigated using solid phase microextraction (SPME) coupled to two-dimensional comprehensive gas chromatography and time-of-flight mass spectrometry (GC ×GC-TOF-MS).

The gas chromatograph (Agilent Technologies 7890B, USA) was equipped with HP-5MS UI capillary column (30 m, 0.25 mm i.d., 0.25-μm film thickness, Agilent USA) coupled via consumable-free modulator with second dimension column VF-17 (1.5 m, 0.1 mm i.d., 0.1-μm film thickness, Agilent USA). Helium at a flow of 1 mL/min was used as a carrier gas. A portion of each independent replication was filtered through 0.22-µm syringe filter. Always 0.5 mL of filtered sample was pipetted into a 10-mL glass vial for headspace analysis and was incubated at 40°C for 10 min in Gerstel autosampler (Gerstel, USA). Then, volatiles were collected for another 10 min at the same temperature on SPME fiber (PDMS/DVB/CAR from Supelco, USA). The injection was applied into a hot (265°C) split/splitless injector. A pulse of 5 psi for 2 min was used together with a split of 1:10 for the sample compoun injection. Oven temperature was increased from an initial 40°C, with a rate of 10°C/min to 160°C, then 25°C/min to 260°C. The second dimension GC oven and modulator followed this temperature program with 5°C and 15°C offset, respectively. The modulation period was 5 s. With a hold time of 5 min, the GC run takes 23 min. Separated compounds were ionized in the ion source of time-of-flight mass spectrometric detector (Pegasus 4D, LECO, USA) at 70 eV in electron impact mode, and full spectral (35–500 Da) information was recorded 100 times per second.

Signals with their unique quantification mass signal-to-noise ratio higher than 150 were aligned in deconvoluted records, creating source data matrix (samples x variables). The input matrix used for PCA and PLS-DA consisted of six biological samples, with aligned volatile features retained after signal filtering as variables. Data were then normalized (constant row sum) and centered log-ratio transformed. After Pareto scaling, data were processed via principal component analysis (PCA) and partial least squares discriminant analysis (PLS-DA) in Simca 18 (Sartorius Stedim, SWE) software.

For tentative identification of compounds, spectral similarity comparison of the measured and deconvoluted spectrum with mass spectra in the National Institute of Standards and Technology mass spectral library (NIST 2017) was performed. Retention indexes were calculated and compared with the NIST MS library to confirm identity.

### Cell surface morphology

The samples were fixed for 24 h in 2.5% glutaraldehyde in 0.2 M cacodylate buffer (pH = 7.0). The samples were then dehydrated using an ethanol series with increasing ethanol concentrations in water. A 10-min incubation was used for each step (35%, 50%, 70%, and 96%). The samples were fixed to a cylinder with electron conductive double-sided adhesive carbon tabs (EM-Tec CT6, Micro to Nano) and coated with gold (10-nm thickness) using a JFC 1300 Auto Fine Coater (JEOL). The samples were then observed with a JSM-IT500HR InTouchScope scanning electron microscope (JEOL) at a voltage of 5 kV with the working distance between 11 and 12 mm.

## RESULTS AND DISCUSSION

### Growth and effect of volume

**Fig 1:**
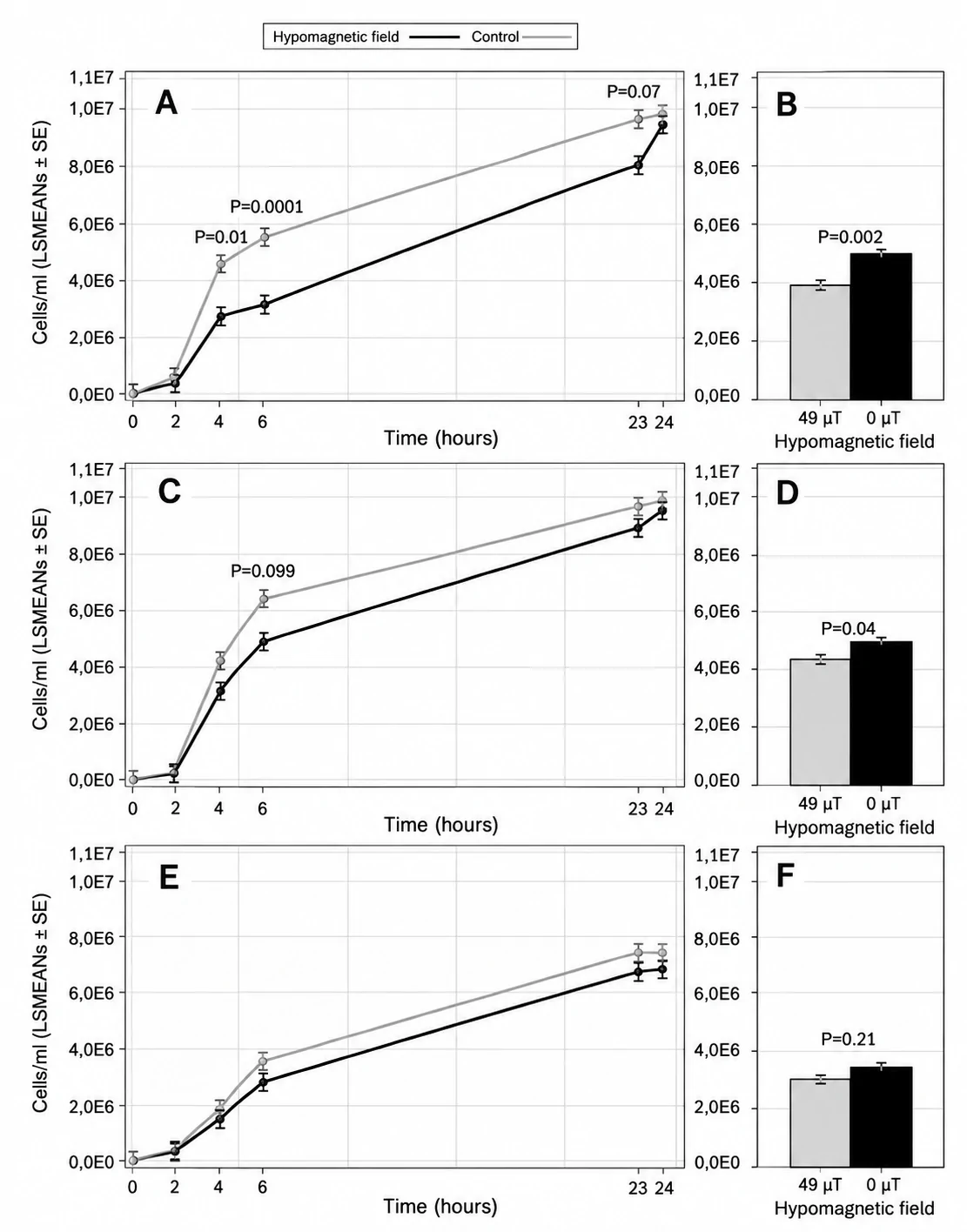
The effect of hypomagnetic field exposure on the growth of the yeast *Saccharomyces cerevisiae* (measured in cells/mL, LSMEANs ± SE) observed in culture volumes of 10 (**A and B**), 75 (**C and D**), and 250 mL (**E and F**), compared with control conditions, with measurements taken at specific time intervals (**A, C, and E**) and summed over the entire cultivation period (**B, D, and F**)

The growth of *Saccharomyces cerevisiae* was significantly influenced by both hypomagnetic exposure and cultivation volume across specific time intervals (F₃₀,₆₀ = 174.95, *P* < 0.0001) as well as over the entire 24-h cultivation period (F₅,₇ = 63.92, *P* < 0.0001). In the smallest tested volume (10 mL), hypomagnetic conditions produced the strongest inhibitory effect, with growth reductions ranging from 16.5% to 42.6% between 4 and 23 h of cultivation (Fig. 1A). In the intermediate volume (75 mL), growth inhibition was also observed, with an average reduction of 23.8% (Fig. 1C). In contrast, no statistically significant inhibition was detected in the largest volume (250 mL) (Fig. 1E). When cumulative growth over the entire cultivation period was evaluated, significant inhibition remained evident in the 10- and 75-mL cultures, but not in the 250-mL cultures (Fig. 1B, D and F).

These results identify cultivation volume as a major modifier of the yeast response to hypomagnetic exposure. The strongest growth inhibition occurred in the smallest static batch cultures, whereas the effect decreased with increasing volume and was no longer statistically detectable in 250-mL cultures. This pattern indicates that magnetic-field deprivation does not act independently of the culture environment but interacts with volume-dependent physiological conditions such as passive gas exchange, metabolite accumulation, nutrient availability, and the transition toward oxygen-limited or fermentative growth. Cultivation volume should therefore be considered a critical experimental parameter in microbial studies of hypomagnetic exposure.

**Fig 2:**
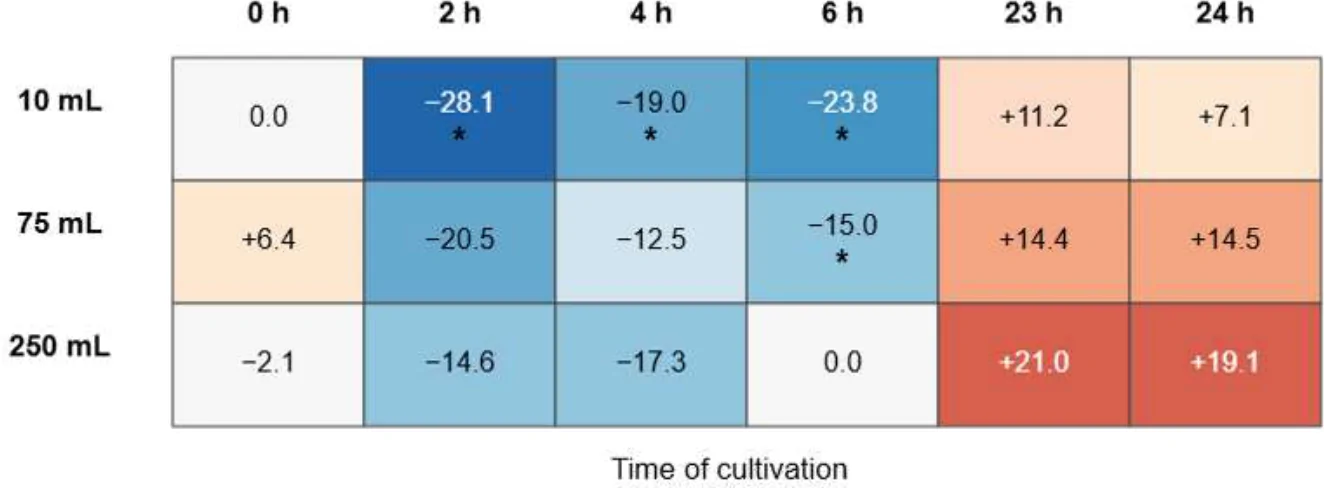
Relative change in budding frequency under hypomagnetic exposure compared with geomagnetic control conditions. Values show percentage change in budding frequency in hypomagnetic cultures relative to the corresponding control at the same time point and cultivation volume. Negative values indicate reduced budding under hypomagnetic exposure. Significant inhibition was detected in 10-mL cultures at 2, 4, and 6 h and in 75-mL cultures at 6 h. No significant inhibition was detected in 250-mL cultures. Asterisks indicate significant differences between hypomagnetic and control treatments at the same time point and volume (*P < 0.05).

Hypomagnetic exposure also affected early cellular development, as shown by reduced budding frequency during the first 6 h of cultivation. In 10-mL cultures, budding was significantly inhibited at 2, 4, and 6 h, with reductions of 28.1%, 19.0%, and 23.8%, respectively, compared with the geomagnetic control. In 75-mL cultures, significant budding inhibition was detected at 6 h, with a reduction of 15.0%. No significant inhibition of budding was detected in 250-mL cultures at any time point. At 23 and 24 h, budding was no longer inhibited, and relative values increased above the control under hypomagnetic exposure; however, these late positive differences were not statistically significant and are therefore interpreted as delayed convergence rather than evidence of growth stimulation (Fig. 2). These results indicate that hypomagnetic exposure transiently delayed early budding in a volume-dependent manner, with the strongest effect occurring in the smallest culture volume. Medium pH decreased during cultivation, consistent with acidification of static yeast batch cultures, but no significant treatment-specific differences were detected between hypomagnetic and geomagnetic control conditions within the tested volumes. Therefore, the observed growth delay and early budding inhibition were not explained by differences in bulk medium pH. Despite these early differences, final cell densities were similar across all treatments, indicating that all cultures ultimately reached stationary phase due to nutrient depletion. However, cultures exposed to hypomagnetic conditions required more time to reach this phase, consistent with their reduced growth rate.

A volume-dependent modulation of magnetic field effects was also observed in our previous experiments using stronger magnetic fields (up to 10 mT) (25). In that study, stimulation of yeast growth and ethanol production decreased as cultivation volume increased. At that time, the phenomenon was partially attributed to potential differences in magnetic field distribution caused by the submerged electromagnetic coil system. In the present study, the experimental setup ensured homogeneous magnetic field conditions across all tested volumes. Therefore, the observed differences in growth inhibition cannot be explained by variations in magnetic field exposure.

Currently, no studies directly address how cultivation volume may influence cellular responses to hypomagnetic conditions. Our results suggest that cultivation volume may act as a modulatory environmental factor affecting the sensitivity of yeast cells to magnetic field deprivation. The present study was designed to characterize phenotypic and metabolic responses to hypomagnetic exposure, whereas detailed mechanistic investigations involving ROS production, ATP dynamics, and intracellular signaling pathways were beyond its scope.

### Volatile compound emission

**Fig 3:**
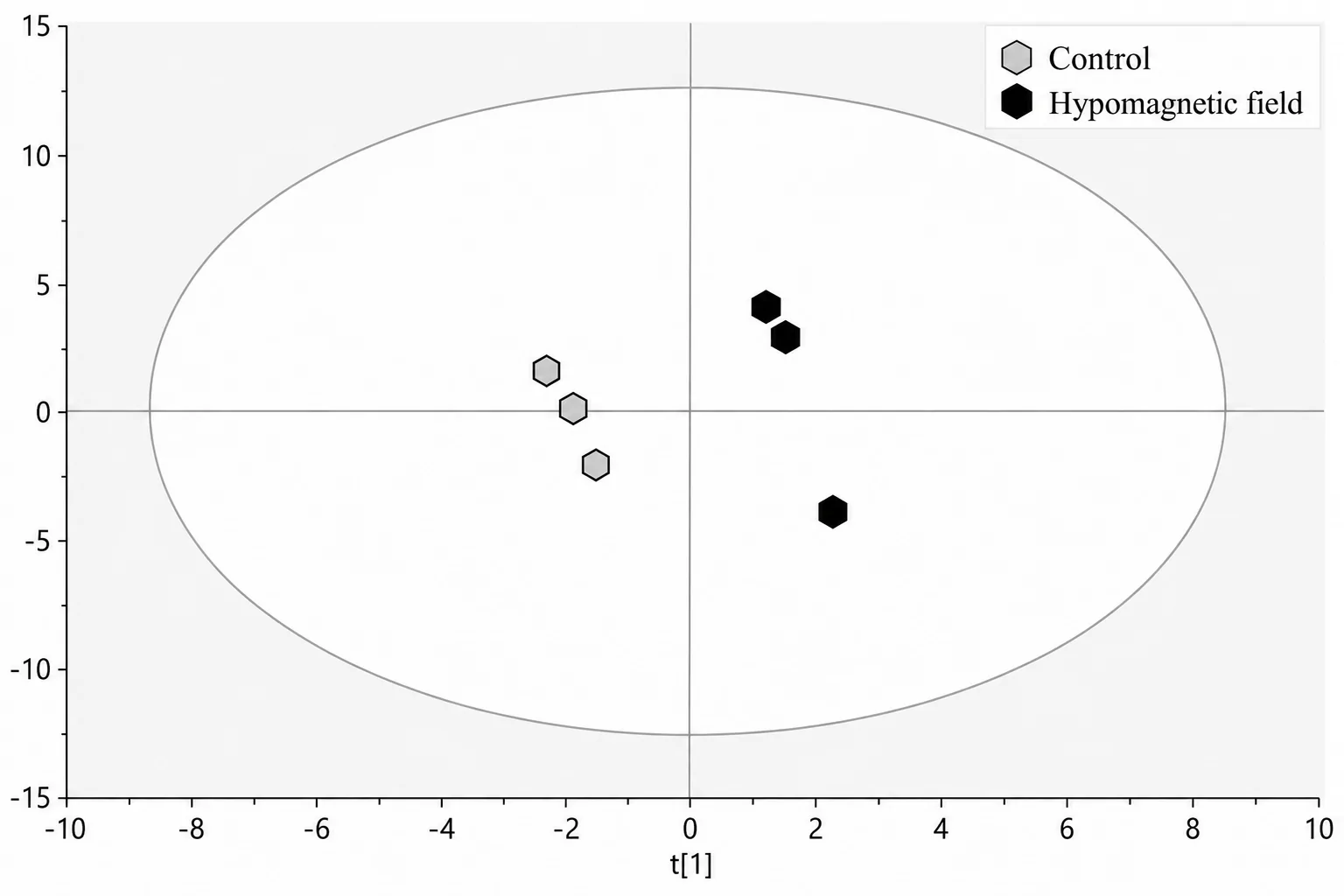
Exploratory partial least squares discriminant analysis (PLS-DA) scores plot of volatile profiles from 10-mL cultures under hypomagnetic and geomagnetic control conditions. The model showed R2X(cum) = 0.48, R2Y(cum) = 1.00, and Q2(cum) = 0.70. Because of the limited number of independent biological replicates, the PLS-DA model was used for exploratory feature prioritization rather than as a validated predictive classifier.

PCA was first used as an unsupervised exploratory analysis to evaluate sample distribution and potential outliers. PLS-DA was then used to identify volatile features contributing to separation between hypomagnetic and geomagnetic control samples. The PLS-DA model showed R2X(cum) = 0.48, R2Y(cum) = 1.00 and Q2(cum) = 0.70 (Fig. 3). Because the number of independent biological replicates was limited and no independent external validation set was available, the PLS-DA model was interpreted as an exploratory feature-prioritization approach rather than as a validated predictive classification model. Therefore, PLS-DA was not used as inferential proof of treatment separation, and volatile differences were interpreted together with VIP values, fold changes, and the observed growth and budding phenotypes.

Acetoin and propanoic acid showed the highest VIP values and were increased by 2.0- and 1.6-fold, respectively, under hypomagnetic exposure (Table 2; Fig. 3). Because both compounds are associated with fermentative carbon metabolism (26, 27), their increase is consistent with a shift in extracellular metabolic output under the static batch conditions used here. Several amino-acid- and lipid-related volatiles also differed between treatments. Isovaleric acid and 2-methylbutanal were increased, consistent with altered branched-chain amino acid catabolism (28, 29), while ethyl octanoate, 1-pentanol, and 2-methylhexanoic acid indicated changes in lipid- or fatty-acid-associated volatile production (30–33).

**TABLE 2:** The effect of hypomagnetic conditions on volatile chemical components emission during yeast *S. cerevisiae* cultivation compared with control (under laboratory conditions)

Chemical compound	VIP value	Produced in a hypomagnetic field X-times more	Interpretation
Acetoin	2.316	2	Intermediate in amino acid metabolism and fermentation; can serve as an alternative carbon source under stress (26)
Propanoic acid	2.052	1.6	By-product of fermentation; can act as a signaling molecule but may also contribute to acid stress (26)
1-Pentanol	1.974	0.7	Product of fatty acid metabolism; its accumulation may indicate stress in lipid metabolism (32)
Isovaleric acid	1.808	1.5	By-product of leucine metabolism; involved in aroma production and may accumulate under nitrogen limitation stress (28)
Styrene	1.670	1.3	Rare in yeast metabolism; typically associated with exogenous exposure; may induce oxidative stress in yeast cells (34)
Ethyl octanoate	1.606	1.3	Ester, derived from fatty acid metabolism; contributes to yeast aroma and indicates lipid metabolic activity (31).
2-Methylhexanoic acid	1.603	0.9	Branched fatty acid; its production may indicate adaptations in lipid metabolism under stress (32).
3-Methylthiopropyl alcohol	1.545	1.3	Metabolite of methionine catabolism; involved in stress response and sulfur metabolism (35) and the synthesis of other sulfur-containing compounds and maintaining redox balance (36)
2-Methylbutanal	1.464	1.4	Derived from isoleucine degradation; can act as a volatile compound under stress (28)
Hexadecane	1.470	1	Long-chain hydrocarbons are not naturally produced by yeast; exogenous exposure may influence membrane integrity (28, 36)

Several compounds commonly linked to cellular stress responses were also elevated. Styrene increased 1.3-fold (VIP = 1.67), and 3-methylthiopropyl alcohol increased 1.3-fold (VIP = 1.55). Although styrene is not a typical product of yeast central metabolism, its presence and relative increase may be associated with altered stress-related metabolism (34), altered sulfur/amino acid metabolism, or exogenous contributions under the tested conditions (35, 36). In parallel, elevated propanoic acid may contribute to acid stress or reflect acid-related regulatory responses during fermentation (37, 38). Notably, these metabolite changes were observed despite similar measured bulk pH trends across treatments, suggesting that the response may involve intracellular or local metabolic regulation rather than only external pH differences.

The volatile changes detected in 10-mL cultures represent extracellular compounds accumulated during cultivation. In the same volume, hypomagnetic exposure produced the strongest growth inhibition and early budding delay, while bulk pH did not differ significantly between treatments. The increase in acetoin and propanoic acid, together with changes in amino acid- and lipid-associated volatiles, therefore supports altered fermentation-associated metabolic output during delayed proliferation. However, these data do not identify the primary intracellular target of hypomagnetic exposure. Compounds with possible exogenous or background origins, such as styrene and hexadecane, were therefore interpreted cautiously.

Together with the growth data, the volatile profile supports the conclusion that hypomagnetic exposure is associated with altered proliferation kinetics and extracellular metabolic output in yeast. These findings are also consistent with our previous observations under stronger magnetic fields (25), suggesting that magnetic field intensity may influence the balance between biomass formation and fermentation-associated metabolism in *S. cerevisiae*.

### Cell surface morphology

**Fig 4:**
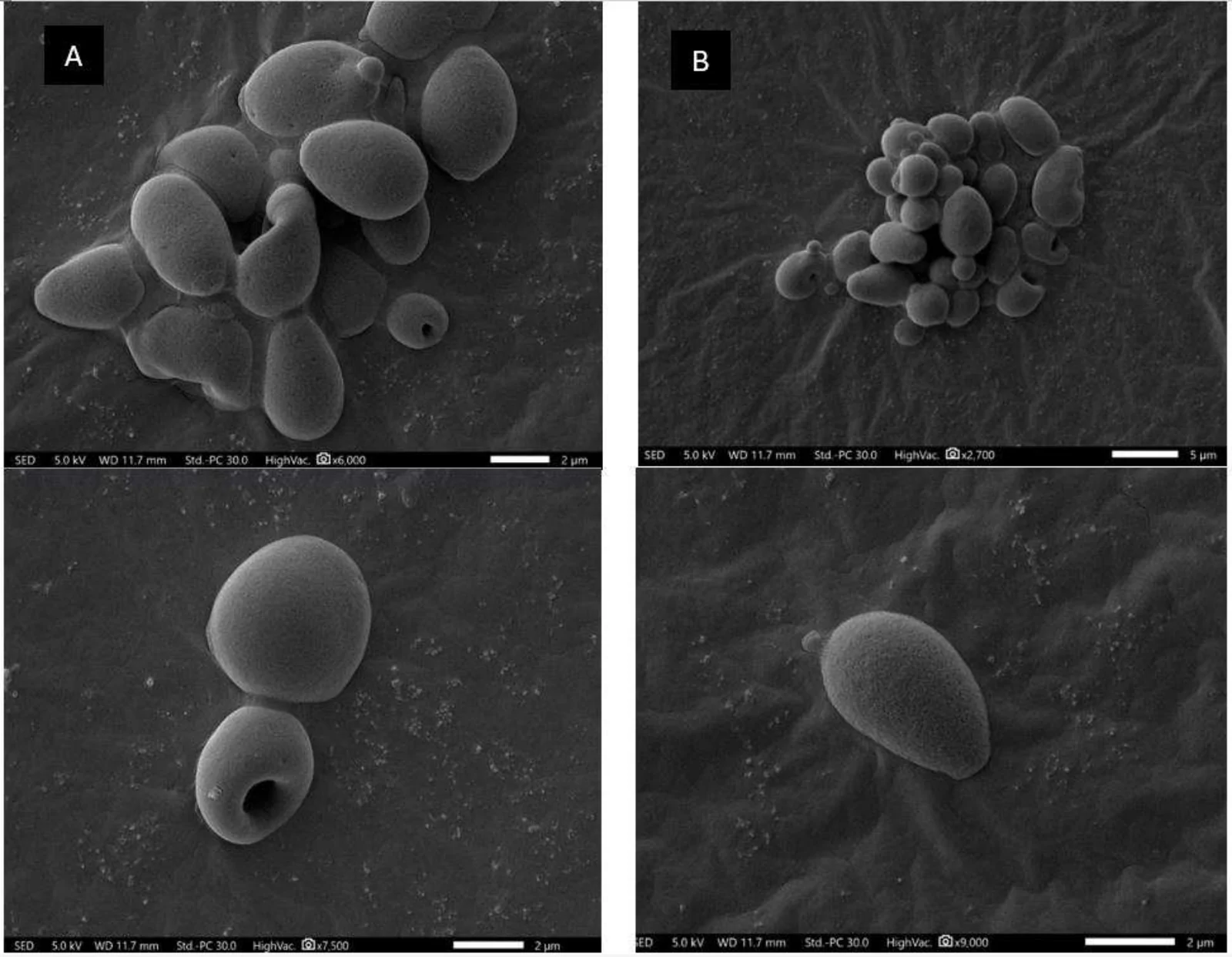
The effect of hypomagnetic conditions (**A**) on the cell-surface morphology of yeast *S. cerevisiae* compared with control (**B**) (in laboratory conditions).

Scanning electron microscopy was used as a qualitative screening approach to determine whether short-term hypomagnetic exposure (24 h) was associated with gross alterations of yeast cell-surface morphology. No clear morphological differences were observed between cells cultivated under hypomagnetic and control conditions, and similar surface irregularities were present in both treatments (Fig. 4). Previous studies have shown that stronger magnetic fields can influence membrane permeability and ion transport in microorganisms (39, 40), and in some cases, such effects have been accompanied by detectable changes in cell morphology or surface structure (41). Evidence for comparable surface- or membrane-associated effects under hypomagnetic conditions remains limited and has been reported mainly in plant and animal systems (10, 42, 43).

This finding constrains the interpretation of the HMF phenotype. The growth delay, altered budding dynamics, and changes in volatile output were not associated with overt cell-surface injury detectable by scanning electron microscopy (SEM), suggesting that the short-term response was primarily growth-kinetic and metabolic rather than cytologically destructive. However, SEM does not directly assess plasma-membrane integrity, permeability, lipid organization, or ion transport; therefore, more subtle membrane-associated effects cannot be excluded and would require targeted functional assays. Whether delayed ultrastructural or membrane-associated changes emerge during prolonged hypomagnetic cultivation remains to be tested.

Membrane integrity staining, permeability measurements, ion transport analysis, fluorescence-based membrane probes, and AFM-based surface-mechanics measurements could help test for subtle membrane-associated effects. In parallel, cell-cycle analysis and quantitative budding assays could clarify whether the primary phenotype reflects delayed proliferative progression rather than surface-level injury. These approaches would help distinguish membrane-associated effects from broader changes in growth physiology, redox state, or fermentative metabolism under hypomagnetic exposure.

### Conclusion

This study shows that short-term hypomagnetic exposure alters growth kinetics and volatile metabolic output in *Saccharomyces cerevisiae* under static batch conditions. The response was strongly dependent on cultivation volume: growth inhibition was most pronounced in small-volume cultures, reduced in intermediate-volume cultures, and not statistically detectable in the largest volume tested. The budding data further support this interpretation by showing significant early inhibition of budding in 10-mL cultures at 2–6 h and in 75-mL cultures at 6 h, while no budding inhibition was detected in 250-mL cultures. This identifies cultivation volume as an important modifier of microbial sensitivity to magnetic-field deprivation.

Volatile profiling further indicated changes in fermentation-associated and amino acid-derived metabolic output under hypomagnetic exposure, whereas SEM analysis did not reveal overt alterations in cell-surface morphology. Together, these findings suggest a growth-kinetic and metabolic response rather than acute structural injury detectable by the methods used here. The molecular basis of this response remains unresolved, and direct measurements of ROS, ATP, redox state, ion transport, membrane function, and cell-cycle progression will be required to define the underlying mechanism.

The main implication of this work is that hypomagnetic field effects in microorganisms should not be evaluated independently of culture physiology. Volume-dependent gas exchange, metabolite accumulation, and growth phase may strongly influence the magnitude of the observed response and should be treated as critical experimental parameters in future studies of microbial magnetic field sensitivity.

## Data Availability

The raw data supporting the findings of this study are available in the Zenodo repository (DOI: https://doi.org/10.5281/zenodo.20702043). The data set includes the raw growth, pH, budding cell, and volatile-compound data used for the analyses presented in the manuscript.
